# Supplementary material for: Floral hosts of leaf-cutter bees (Megachilidae) in a biodiversity hotspot revealed by pollen DNA metabarcoding of historic specimens
Source: PLoS One. 2021 Jan 21;16(1):e0244973. doi: 10.1371/journal.pone.0244973 (PMC7819603; doi:10.1371/journal.pone.0244973)
Supplement: S1 Table — Collection information, such as the date, province, GPS coordinates and nearest town are given for each sample, where available. The new province and town designations are given in brackets where they have been changed. (DOCX) [file pone.0244973.s001.docx]

**S1 Table. The National Insect Collection, ARC, South Africa Collection information of *Megachile karooensis* bee specimens from which pollen was collected for this study.** Collection information**,** such as the date, province, GPS coordinates and nearest town are given for each sample, where available. The new province and town designations are given in brackets where they have been changed.

| **Bee collection identifier^1^** | **Pollen sample identifier** | **Bee collection date** | **Province^2^** | **GPS** | **Bee collection description** |
| --- | --- | --- | --- | --- | --- |
| HYMA06233 | b1 | 19.11.1982 | Western Cape | 33.23S 19.27E | Mitchell’s Pass near Ceres |
| HYMA06345 | b2 | 09.09.1987 | Western Cape | 31.13S 19.12E | Brandkop |
| HYMA05853/1 | b3 | 05-07.09.1987 | Western Cape | 32.08S 19.14E | Clanwilliam District, Biedouw Valley |
| HYMA05853/2 | b4 | 05-07.09.1987 | Western Cape | 32.08S 19.14E | Clanwilliam District, Biedouw Valley |
| HYMA05852 | b5 | 27.09.1990 | Western Cape | 32.08S 19.14E | Clanwilliam District, Biedouw Valley |
| HYMA05853/3 | b6 | 05-07.09.1987 | Western Cape | 32.08S 19.14E | Clanwilliam District, Biedouw Valley |
| HYMA06359 | b7 | 01.10.1990 | Western Cape | 30.09S 17.59E | Dassiefontein Farm near Kammieskroon |
| HYMA22079 | b8 | 05-07.09.1987 | Western Cape | 32.08S 19.14E | Clanwilliam District, Biedouw Valley |
| HYMA29217 | b9 | 05-07.09.1987 | Western Cape | 32.08S 19.14E | Clanwilliam District, Biedouw Valley |
| HYMA29218 | b10 | 05-07.09.1987 | Western Cape | 32.08S 19.14E | Clanwilliam District, Biedouw Valley |
| HYMA29219 | b11 | 05-07.09.1987 | Western Cape | 32.08S 19.14E | Clanwilliam District, Biedouw Valley |
| HYMA29220 | b12 | 05-07.09.1987 | Western Cape | 32.08S 19.14E | Clanwilliam District, Biedouw Valley |
| HYMA29221 | b13 | 05-07.09.1987 | Western Cape | 32.08S 19.14E | Clanwilliam District, Biedouw Valley |
| HYMA29222 | b14 | 05-07.09.1987 | Western Cape | 32.08S 19.14E | Clanwilliam District, Biedouw Valley |
| HYMA29223 | b15 | 05-07.09.1987 | Western Cape | 32.08S 19.14E | Clanwilliam District, Biedouw Valley |
| HYMA29224 | b16 | 05-07.09.1987 | Western Cape | 32.08S 19.14E | Clanwilliam District, Biedouw Valley |
| HYMA29225 | b17 | 05-07.09.1987 | Western Cape | 32.08S 19.14E | Clanwilliam District, Biedouw Valley |
| HYMA29226 | b18 | 05-07.09.1987 | Western Cape | 32.08S 19.14E | Clanwilliam District, Biedouw Valley |
| HYMA29227 | b19 | 05-07.09.1987 | Western Cape | 32.08S 19.14E | Clanwilliam District, Biedouw Valley |
| HYMA29228 | b20 | 03.09.1987 | Western Cape | NA | Namaqualand (Grid 2917DB) |

^1^Bees selected for pollen removal were obtained from the National Insect Collection housed at the ARC’s Biosystematics, Pretoria, South Africa.

^2^South Africa has renamed some of their provinces and towns. The original collection province and towns, as well as the new names in brackets, are given.
