# Supplementary material for: Floral hosts of leaf-cutter bees (Megachilidae) in a biodiversity hotspot revealed by pollen DNA metabarcoding of historic specimens
Source: PLoS One. 2021 Jan 21;16(1):e0244973. doi: 10.1371/journal.pone.0244973 (PMC7819603; doi:10.1371/journal.pone.0244973)
Supplement: S2 Table — Collection information, such as the date, province, GPS coordinates and nearest town are given for each sample, where available. The new province and town designations are given in brackets where they have been changed. (DOCX) [file pone.0244973.s002.docx]

**S2 Table. Collection information from the National Insect Collection, ARC, South Africa, of *Megachile murina* bee specimens from which pollen was collected for the Succulent Karoo group in this study.** Collection information**,** such as the date, province, GPS coordinates and nearest town are given for each sample, where available. The new province and town designations are given in brackets where they have been changed.

| **Bee collection identifier** | **Pollen sample identifier** | **Bee collection date** | **Province** | **GPS** | **Bee collection description** |
| --- | --- | --- | --- | --- | --- |
| HYMA06346 | d1 | 16.11.1984 | Western Cape | 32.08S 19.01E | Pakhuis Pass |
| HYMA06342 | d2 | 11.09.1987 | Western Cape | 30.10S 18.01E | Kamiesberg |
| HYMA06233/1 | d3 | 19.11.1982 | Western Cape | 33.23S 19.27E | Mitchell’s Pass near Ceres |
| HYMA06233/2 | d4 | 19.11.1982 | Western Cape | 33.23S 19.27E | Mitchell’s Pass near Ceres |
| HYMA06233/3 | d5 | 19.11.1982 | Western Cape | 33.23S 19.27E | Mitchell’s Pass near Ceres |
| HYMA06316/1 | d6 | 16.12.1988 | Western Cape | 33.19S 21.25E | North of Seweweekspoort |
| HYMA06316/2 | d7 | 16.12.1988 | Western Cape | 33.19S 21.25E | North of Seweweekspoort |
| HYMA06316/3 | d8 | 16.12.1988 | Western Cape | 33.19S 21.25E | North of Seweweekspoort |
| HYMA06343/1 | d9 | 07.09.1987 | Western Cape | 32.08S 19.02E | Pakhuis Pass |
| HYMA06316/4 | d10 | 16.12.1988 | Western Cape | 33.19S 21.25E | North of Seweweekspoort |
| HYMA06343/2 | d11 | 07.09.1987 | Western Cape | 32.08S 19.02E | Pakhuis Pass |
| HYMA05855 | d12 | 01.10.1990 | Western Cape | 30.09S 17.59E | Dassiefontein Farm near Kamieskroon |
| HYMA29232 | d13 | 07.09.1987 | Western Cape | 32.08S 19.02E | Pakhuis Pass |
| HYMA29233 | d14 | 07.09.1987 | Western Cape | 32.08S 19.02E | Pakhuis Pass |
| HYMA29234 | d15 | 07.09.1987 | Western Cape | 32.08S 19.02E | Pakhuis Pass |
| HYMA22083 | d16 | 27.09.1987 | Western Cape | NA | Namaqualand (Grid 2917 DB) |
| HYMA29235 | d17 | 27.09.1987 | Western Cape | NA | Namaqualand (Grid 2917 DB) |
| HYMA29236 | d18 | 16.09.1987 | Western Cape | NA | Namaqualand (Grid 2917 DB) |
| HYMA22085 | d19 | 03.09.1987 | Western Cape | NA | Namaqualand (Grid 2917 DB) |
| HYMA29237 | d20 | 03.09.1987 | Western Cape | NA | Namaqualand (Grid 2917 DB) |
| HYMA22086 | d21 | 25.09.1987 | Western Cape | NA | Namaqualand (Grid 2917 DB) |
| HYMA22087 | d22 | 17.11.1982 | Western Cape | 33.48S 20.12E | Ouberg Pass, 24 km North East of Montagu |
| HYMA29238 | d23 | 17.11.1982 | Western Cape | 33.48S 20.12E | Ouberg Pass, 24 km North East of Montagu |
| HYMA29239 | d24 | 17.11.1982 | Western Cape | 33.48S 20.12E | Ouberg Pass, 24 km North East of Montagu |
| HYMA29240 | d25 | 17.11.1982 | Western Cape | 33.48S 20.12E | Ouberg Pass, 24 km North East of Montagu |
| HYMA29241 | d26 | 17.11.1982 | Western Cape | 33.48S 20.12E | Ouberg Pass, 24 km North East of Montagu |
| HYMA29231 | d27 | 17.11.1982 | Western Cape | 33.48S 20.12E | Ouberg Pass, 24 km North East of Montagu |
