# Supplementary material for: Floral hosts of leaf-cutter bees (Megachilidae) in a biodiversity hotspot revealed by pollen DNA metabarcoding of historic specimens
Source: PLoS One. 2021 Jan 21;16(1):e0244973. doi: 10.1371/journal.pone.0244973 (PMC7819603; doi:10.1371/journal.pone.0244973)
Supplement: S3 Table — Available information regarding the specimen collection, such as date, province, GPS coordinates and collection locality are given for each sample. (DOCX) [file pone.0244973.s003.docx]

**S3 Table. The National Insect Collection, ARC, South Africa Collection information of *Megachile maxillosa* bee specimens from which pollen was collected for the widespread group in this study.** Available information regarding the specimen collection**,** such as date, province, GPS coordinates and collection locality are given for each sample.

| **Bee collection identifier** | **Pollen sample identifier** | **Bee collection date** | **Province** | **GPS** | **Bee collection description** |
| --- | --- | --- | --- | --- | --- |
| HYMA06176 | c1 | 10.1979 | Limpopo | NA | Mogoto Reserve near Zebediela |
| HYMA06202 | c2 | 11.1976 | North West | NA | Buffelspoort near Rustenburg |
| HYMA06232 | c3 | 24.10.1975 | Northern Cape | NA | Upington |
| HYMA21729 | c4 | 19.10.1980 | NA | NA | Pumulani |
| HYMA21731 | c5 | 19.12.2003 | KwaZulu-Natal | NA | Bisley Nature Reserve, Pietermaritzburg |
| HYMA06223 | c6 | 17.11.1972 | Limpopo | NA | Rooiberg, east of Thabazimbi |
| HYMA05911 | c7 | 25.02.1993 | Free State | 27.40S 25.45E | Sandveld Nature Reserve |
| HYMA06195 | c8 | 22.10.1914 | Free State | NA | Bloemfontein |
| HYMA06194 | c9 | 17.10.1914 | Free State | NA | Bloemfontein |
| HYMA06222 | c10 | 19.01.1984 | Mpumalanga | 24.59S 31.55E | Skukuza, Kruger National Park |
| HYMA21737 | c11 | 22-23.02.1993 | Free State | 27.15S 27.41E | Koppies Dam Nature Reserve |
| HYMA06226 | c12 | 04.1975 | North West | NA | Buffelspoort |
| HYMA06205/1 | c13 | 31.03.1972 | Limpopo | NA | Ellisras |
| HYMA06205/2 | c14 | 31.03.1972 | Limpopo | NA | Ellisras |
| HYMA06205/3 | c15 | 31.03.1972 | Limpopo | NA | Ellisras |
| HYMA06175 | c16 | 01.11.1969 | Transvaal^1^ | NA | Breedsnek Pass |
| HYMA06184 | c17 | 06.04.1962 | Limpopo | NA | Ellisras |
| HYMA06176 | c18 | 10.1979 | Limpopo | NA | Mogoto Reserve near Zebediela |
| HYMA21741 | c19 | 17.02.1981 | Gauteng | 25.24S 28.06E | Soutpan, Pretoria district |
| HYMA06308 | c20 | 10.12.1978 | Northern Cape | NA | Olifantshoek |
| HYMA21742 | c21 | 22-23.02.1993 | Free State | 27.15S 27.41E | Koppies Dam Nature Reserve |
| HYMA06179 | c22 | 14.03.1969 | Eastern Cape | NA | Graaff Reinet |
| HYMA06224 | c23 | 02.12.1981 | Limpopo | 24.13S 29.30E | Chuniespoort |
| HYMA06305/1 | c24 | 18-29.10.1989 | Northern Cape | 26.25S 20.37E | Kalahari Gemsbok Park, Twee Rivieren |
| HYMA06305/2 | c25 | 18-29.10.1989 | Northern Cape | 26.25S 20.37E | Kalahari Gemsbok Park, Twee Rivieren |
| HYMA06305/3 | c26 | 18-29.10.1989 | Northern Cape | 26.25S 20.37E | Kalahari Gemsbok Park, Twee Rivieren |
| HYMA06200/1 | c27 | 10.1978 | Limpopo | NA | Nylsvley Nature Reserve |
| HYMA06335 | c28 | 19.12.1987 | Limpopo | 23.45S 27.49E | D’Nyala Nature Reserve, Ellisras District |
| HYMA06200/2 | c29 | 10.1978 | Limpopo | NA | Nylsvley Nature Reserve |
| HYMA06200/3 | c30 | 10.1978 | Limpopo | NA | Nylsvley Nature Reserve |
| HYMA06181 | c31 | 24-28.11.1980 | Limpopo | 24.37S 27.23E | Ben Alberts Nature Reserve, Thabazimbi |
| HYMA06200/4 | c32 | 10.1978 | Limpopo | NA | Nylsvley Nature Reserve |

^1^ The borders of South Africa’s provinces have changed, and with it also the province names. Breedsnek Pass used to fall within one province, Transvaal, but now stretches through Gauteng and the North West Province. Without GPS coordinates, it was not possible to ascertain in which of the renamed provinces the sample was collected.
