# Supplementary material for: Floral hosts of leaf-cutter bees (Megachilidae) in a biodiversity hotspot revealed by pollen DNA metabarcoding of historic specimens
Source: PLoS One. 2021 Jan 21;16(1):e0244973. doi: 10.1371/journal.pone.0244973 (PMC7819603; doi:10.1371/journal.pone.0244973)
Supplement: S4 Table — Available information regarding the specimen collection, such as date, province, GPS coordinates and collection locality are given for each sample. (DOCX) [file pone.0244973.s004.docx]

**S4 Table. Collection information from the National Insect Collection, ARC, South Africa, of *Megachile felina* bee specimens from which pollen was collected for the Savanna group in this study.** Available information regarding the specimen collection**,** such as date, province, GPS coordinates and collection locality are given for each sample.

| **Bee collection identifier** | **Pollen sample identifier** | **Bee collection date** | **Province** | **GPS** | **Bee collection description** |
| --- | --- | --- | --- | --- | --- |
| HYMA06277/1 | a1 | 25-26.10.1984 | Mpumalanga | 24.32S 30.47E | Blyderivierspoort Nature Reserve |
| HYMA06277/2 | a2 | 25-26.10.1984 | Mpumalanga | 24.32S 30.47E | Blyderivierspoort Nature Reserve |
| HYMA06277/3 | a3 | 25-26.10.1984 | Mpumalanga | 24.32S 30.47E | Blyderivierspoort Nature Reserve |
| HYMA06277/4 | a4 | 25-26.10.1984 | Mpumalanga | 24.32S 30.47E | Blyderivierspoort Nature Reserve |
| HYMA06301 | a5 | 09.03.1990 | Limpopo | 22.14S 29.59E | Near Beitbridge |
| HYMA06272 | a6 | 08-12.12.1989 | Limpopo | 23.45S 27.49E | D’Nyala Nature Reserve, Ellisras District |
| HYMA06297/1 | a7 | 07.03.1990 | Limpopo | 30.03S 22.23E | Messina Nature Reserve |
| HYMA06297/2 | a8 | 07.03.1990 | Limpopo | 30.03S 22.23E | Messina Nature Reserve |
| HYMA06156 | a9 | 10-11.12.1979 | Limpopo | 24.39S 28.42E | Nylsvlei Nature Reserve |
| HYMA06146 | a10 | 12.1974 | North West | NA | Mokopane |
| HYMA06464 | a11 | 01.1987 | Gauteng | 25.24S 28.06E | Soutpan, Pretoria district |
| HYMA22025 | a12 | 04.1970 | Limpopo | NA | Letsitele |
| HYMA22045 | a13 | 24.01.1985 | Limpopo | 22.36S 31.17E | Machayi Pan 418 m, Kruger National Park |
| HYMA29229 | a14 | 24.01.1985 | Limpopo | 22.36S 31.17E | Machayi Pan 418 m, Kruger National Park |
| HYMA29230 | a15 | 20-24-01.1985 | Limpopo | 22.26S 31.12E | Pafuri 264 m, Kruger National Park |
| HYMA22063 | a16 | 20-24.01.1985 | Limpopo | 22.26S 31.12E | Pafuri 264 m, Kruger National Park |
| HYMA22076 | a17 | 12.01.1966 | Limpopo | NA | Shipudza (Grid 2230 BD), Kruger National Park |
