# Supplementary material for: Floral hosts of leaf-cutter bees (Megachilidae) in a biodiversity hotspot revealed by pollen DNA metabarcoding of historic specimens
Source: PLoS One. 2021 Jan 21;16(1):e0244973. doi: 10.1371/journal.pone.0244973 (PMC7819603; doi:10.1371/journal.pone.0244973)
Supplement: S5 Table — Available information regarding the specimen collection, such as date, province, GPS coordinates and collection locality are given for each sample. (DOCX) [file pone.0244973.s005.docx]

**S5 Table. Collection information from the National Insect Collection, ARC, South Africa, of *Megachile niveofasciata* bee specimens from which pollen was collected for the widespread group in this study.** Available information regarding the specimen collection**,** such as date, province, GPS coordinates and collection locality are given for each sample.

| **Bee collection identifier** | **Pollen sample identifier** | **Bee collection date** | **Province** | **GPS** | **Bee collection description** |
| --- | --- | --- | --- | --- | --- |
| HYMA29242 | f1 | 07.10.1987 | Northern Cape | NA | Richtersveld National Park, Road Rhubus-Ochta near Vyfsusters-Mt (Grid 2816 BB) |
| HYMA29243 | f2 | 07.10.1987 | Northern Cape | NA | Richtersveld National Park, Road Rhubus-Ochta near Vyfsusters-Mt (Grid 2816 BB) |
| HYMA29244 | f3 | 02.02.2000 | Northern Cape | 29.04S 19.24E | 8 km North East of Pofadder |
| HYMA29245 | f4 | 02.02.2000 | Northern Cape | 29.04S 19.24E | 8 km North East of Pofadder |
| HYMA06323/1 | f5 | 09.12.1990 | Western Cape | 33.16S 19.43E | Verlorenvlei near Ceres |
| HYMA06323/2 | f6 | 09.12.1990 | Western Cape | 33.16S 19.43E | Verlorenvlei near Ceres |
| HYMA06323/3 | f7 | 09.12.1990 | Western Cape | 33.16S 19.43E | Verlorenvlei near Ceres |
| HYMA06323/4 | f8 | 09.12.1990 | Western Cape | 33.16S 19.43E | Verlorenvlei near Ceres |
| HYMA06352 | f9 | 17.11.1984 | Western Cape | 31.59S 19.14E | Doringbos |
| HYMA06365 | f10 | 02.11.1992 | Western Cape | 29.27S 17.03E | Kwakanap Road on Kleinsee Road 20 km, South East of Port Nolloth |
