# Supplementary material for: Floral hosts of leaf-cutter bees (Megachilidae) in a biodiversity hotspot revealed by pollen DNA metabarcoding of historic specimens
Source: PLoS One. 2021 Jan 21;16(1):e0244973. doi: 10.1371/journal.pone.0244973 (PMC7819603; doi:10.1371/journal.pone.0244973)
Supplement: S6 Table — Groups were set as bee species. The only two bee species differing significantly from others were the two widespread species, M. venusta and M. niveofasciata. (DOCX) [file pone.0244973.s006.docx]

| **S6 Table. Collection information of *Megachile venusta* bee specimens used for pollen sample collection in this study**. Bees were obtained from the National Insect Collection housed at the ARC’s Biosystematics, Pretoria, South Africa. Collection information, such as the date, province, GPS coordinates and nearest town are given for each sample, where available. | | | | | |
| --- | --- | --- | --- | --- | --- |
| **Bee collection identifier^1^** | **Sample identifier in this study** | **Bee collection date** | **Province^2^** | **GPS** | **Bee collection description** |
| HYMA05682 | v1 | 10.12.1916 | Transvaal (Gauteng) | 25.43S 28.11E | Pretoria |
| HYMA05679 | v2 | 30.10.1914 | Orange Free State (Free State) | 29.07S27.28E | Modderpoort |
| HYMA05680 | v3 | 05.11.1914 | Orange Free State (Free State) | 29.27S26.13E | Bloemfontein |
| HYMA05677/1 | v4 | 18.10.1921 | North West | 27.32S 24.48E | Taung |
| HYMA05678 | v5 | 11.1921 | Transvaal (Mpumalanga) | 25.28S 30.59E | Nelspruit (Mbombela) |
| HYMA05677/2 | v6 | 18.10.1921 | North West | 27.32S 24.48E | Taung |
| HYMA27297/1 | v7 | 12.1948 | Eastern Cape | 33.50S 25.34E | Redhouse near Port Elizabeth |
| HYMA05723 | v8 | 21.05.1969 | Transvaal(Mpumalanga) | 25.21S 31.53E | Kruger National Park at Crocodile bridge |
| HYMA05505 | v9 | 23.09.1962 | Transvaal (North West) | 25.39S 26.41E | Swartruggens |
| HYMA05503 | v10 | 5.03.1963 | Natal (KwaZulu-Natal) | 29.00S 29.53E | Estcourt |
| HYMA05769 | v11 | 10.02.1977 | Northern Cape | 27.27S 23.26E | Kuruman |
| HYMA05520 | v12 | 03.01.1970 | Transvaal (Gauteng) | 25.56S 28.13E | Olifantsfontein |
| HYMA05608 | v13 | 11.12.1977 | Transvaal (North West) | 26.42S 27.05E | Potchefstroom (Tlokwe) |
| HYMA05804/1 | v14 | 29.10.1985 | Transvaal (Gauteng) | 25.45S 28.12E | Pretoria at Gardens of Union Building |
| HYMA05804/2 | v15 | 29.10.1986 | Transvaal (Gauteng) | 25.45S 28.12E | Pretoria at Gardens of Union Building |
| HYMA05890 | v16 | 10.11.1981 | Natal (KwaZulu-Natal) | 28.55S 29.14E | Cathedral Peak Forestry Area |
| HYMA27298 | v17 | 11.01.1993 | Gauteng | 25.41S 28.18E | Roodeplaat Research Station |
| HYMA05911 | v18 | 24-25.02.1993 | Free State | 27.40S 25.45E | Sandveld Nature Reserve |
| HYMA27299 | v19 | 24-25.02.1993 | Free State | 27.40S 25.45E | Sandveld Nature Reserve |
| HYMA27300 | v20 | 09.09.2007 | Northern Cape | 28.07S 17.00E | Richtersveld National Park at Hand of God |
| HYMA27301 | v21 | 19.01.2004 | Northern Cape | 27.13S 22.55E | 4 km West of Hotazel |
| HYMA27302 | v22 | 19.09.2005 | Western Cape | 32.12S 18.53E | South of Clanwilliam |

^1^National Insect Collection’s (ARC, Pretoria) unique identifiers.

^2^South Africa has re-divided and renamed some of their provinces and towns. The original collection province and towns are given with the new names provided in brackets.
