# Supplementary material for: Floral hosts of leaf-cutter bees (Megachilidae) in a biodiversity hotspot revealed by pollen DNA metabarcoding of historic specimens
Source: PLoS One. 2021 Jan 21;16(1):e0244973. doi: 10.1371/journal.pone.0244973 (PMC7819603; doi:10.1371/journal.pone.0244973)
Supplement: S7 Table — References from which data were sourced are given, as well as any additional information available. (DOCX) [file pone.0244973.s007.docx]

**S7 Table:** **A list of plant species and families on which visits from the six studied bee species have been recorded.** References from which data were sourced are given, as well as any additional information available.

| **Bee species** | **Plant species visited** | **Plant family visited** | **Additional information and references** |
| --- | --- | --- | --- |
| *Megachile karooensis* | *Anchusa capensis* | Boraginaceae | Agricultural Research Council, 2016 |
|  | *Aspalathus linearis* | Fabaceae | Agricultural Research Council, 2016; Eardley, 2012 |
|  | *Aspalathus pulicifolia* | Fabaceae | Agricultural Research Council, 2016; Eardley, 2012 |
|  | *Aspalathus spinescens* | Fabaceae | Agricultural Research Council, 2016; Eardley, 2012 |
|  | *Aspalathus* sp. | Fabaceae | Eardley, 2012 |
|  | *Blepharis extenuata* | Acanthaceae | Agricultural Research Council, 2016; Eardley, 2012 |
|  | *Disa filicornis* | Orchidaceae | Agricultural Research Council, 2016; Eardley, 2012 |
|  | *Hermannia trifurca* | Malvaceae | Agricultural Research Council, 2016 |
|  | *Hermbstaedtia glauca* | Amaranthaceae | Agricultural Research Council, 2016; Eardley, 2012 |
|  | *Lebeckia pungens* | Fabaceae | Agricultural Research Council, 2016; Eardley, 2012 |
|  | *Lebeckia sericea* | Fabaceae | Agricultural Research Council, 2016; Eardley, 2012 |
|  | *Lebeckia spinescens* | Fabaceae | Agricultural Research Council, 2016; Eardley, 2012 |
|  | *Moraea tripetala* | Iridaceae | Agricultural Research Council, 2016; Eardley, 2012 |
|  | *Nemesia* sp. | Scrophulariaceae | Agricultural Research Council, 2016; Eardley, 2012 |
|  | *Pelargonium capitatum* | Geraniaceae | Gess and Gess, 2014 |
|  | *Pelargonium* sp. | Geraniaceae | Agricultural Research Council, 2016 |
|  | *Polygala virgata* | Polygalaceae | Agricultural Research Council, 2016 |
|  | *Polymita albiflora* | Aizoaceae | Agricultural Research Council, 2016; Eardley, 2012 |
|  | *Pteronia incana* | Asteraceae | Agricultural Research Council, 2016; Eardley, 2012 |
|  | *Wiborgia monoptera* | Fabaceae | Agricultural Research Council, 2016; Eardley, 2012 |
|  | *Zygophyllum meyeri* | Zygophyllaceae | Agricultural Research Council, 2016; Eardley, 2012 |
|  | *Zygophyllum retrofractum* | Zygophyllaceae | Agricultural Research Council, 2016; Eardley, 2012 |
|  | - | Fabaceae | Particularly Papilionaceae, Eardley, 2012 |
| *Megachile murina* | *Albuca sp.* | Asparagaceae | Agricultural Research Council, 2016 |
|  | *Anchusa capensis* | Boraginaceae | Agricultural Research Council, 2016 |
|  | *Aridaria brevicarpa* | Aizoaceae | Agricultural Research Council, 2016; Eardley, 2012 |
|  | *Aspalathus chortophila* | Fabaceae | Agricultural Research Council, 2016; Eardley, 2012 |
|  | *Aspalathus linearis* | Fabaceae | Agricultural Research Council, 2016; Eardley, 2012 |
|  | *Aspalathus pulicifolia* | Fabaceae | Agricultural Research Council, 2016; Eardley, 2012 |
|  | *Aspalathus spinescens* | Fabaceae | Agricultural Research Council, 2016; Eardley, 2012 |
|  | *Aspalathus* sp. | Fabaceae | Gess and Gess, 2014 |
|  | *Ballota africana* | Lamiaceae | Agricultural Research Council, 2016 |
|  | *Chrysanthemum sp.* | Asteraceae | Eardley, 2012 |
|  | *Hermannia disermifolia* | Malvaceae | Agricultural Research Council, 2016 |
|  | *Herrea* sp. | Aizoaceae | Agricultural Research Council, 2016 |
|  | *Hirpicium alienatum* | Asteraceae | Agricultural Research Council, 2016; Eardley, 2012 |
|  | *Lebeckia pungens* | Fabaceae | Agricultural Research Council, 2016; Eardley, 2012 |
|  | *Lebeckia sericea* | Fabaceae | Agricultural Research Council, 2016; Eardley, 2012 |
|  | *Lebeckia simsiana* | Fabaceae | Agricultural Research Council, 2016 |
|  | *Lebeckia* sp. | Fabaceae | Eardley, 2012 |
|  | *Lotononis bainesii* | Fabaceae | Agricultural Research Council, 2016; Gess and Gess, 2014 |
|  | *Polygala virgata* | Polygalaceae | Agricultural Research Council, 2016; Gess and Gess, 2014 |
|  | *Polygala* sp. | Polygalaceae | Gess and Gess, 2014 |
|  | *Polymita albiflora* | Aizoaceae | Agricultural Research Council, 2016 |
|  | *Prenia pallens* | Aizoaceae | Agricultural Research Council, 2016 |
|  | *Sarcocaulon crassicaule* | Geraniaceae | Agricultural Research Council, 2016; Eardley, 2012 |
|  | *Stachys aurea* | Lamiaceae | Single specimen, Agricultural Research Council, 2016; Gess and Gess, 2014 |
|  | *Wahlenbergia sp.* | Campanulaceae | Eardley, 2012 |
|  | *Wiborgia monoptera* | Fabaceae | Agricultural Research Council, 2016; Eardley, 2012 |
|  | *Wiborgia* sp. | Fabaceae | Agricultural Research Council, 2016; Gess and Gess, 2014 |
|  | *Zygophyllum divaricatum* | Zygophyllaceae | Agricultural Research Council, 2016 |
|  | *Zygophyllum meyeri* | Zygophyllaceae | Agricultural Research Council, 2016; Eardley, 2012 |
|  | *-* | Fabaceae | Close association, Papilionaceae Agricultural Research Council, 2016; Gess and Gess, 2014 |
|  | *-* | Lamiaceae | Single female, Gess and Gess, 2014 |
| *Megachile felina* | *Cleome angustifolia* | Cleomaceae | Agricultural Research Council, 2016 |
|  | *Crotalaria argyraea* | Fabaceae | Agricultural Research Council, 2016; Eardley, 2012 |
|  | *Crotalaria podocarpa* | Fabaceae | Agricultural Research Council, 2016 |
|  | *Crotalaria* sp. | Fabaceae | Gess and Gess, 2014 |
|  | *Eulophia streptopetala* | Orchidaceae | Agricultural Research Council, 2016 |
|  | *Otoptera burchellii* | Fabaceae | Agricultural Research Council, 2016; Eardley, 2012 |
|  | *Tephrosia oxygona* | Fabaceae | Agricultural Research Council, 2016; Gess and Gess, 2014 |
|  | - | Asteraceae | Agricultural Research Council, 2016; Eardley, 2012 |
| *Megachile maxillosa* | *Acacia horrida* | Fabaceae | Eardley, 2012 |
|  | *Acacia karroo* | Fabaceae | Agricultural Research Council, 2016 |
|  | *Acacia nilotica* | Fabaceae | Eardley, 2012 |
|  | *Acacia senegal* | Fabaceae | Agricultural Research Council, 2016 |
|  | *Adenolobus pechuelii* | Fabaceae | Agricultural Research Council, 2016 |
|  | *Asclepias buchenaviana* | Asclepiadaceae | Agricultural Research Council, 2016; Eardley, 2012 |
|  | *Blepharis capensis* | Acanthaceae | Agricultural Research Council, 2016 |
|  | *Blepharis* sp. | Acanthaceae | Gess and Gess, 2014 |
|  | *Cleome elegantissima* | Cleomaceae | Agricultural Research Council, 2016 |
|  | *Cleome suffruticosa* | Cleomaceae | Agricultural Research Council, 2016 |
|  | *Cleome* sp. | Cleomaceae | Gess and Gess, 2014 |
|  | *Crotalaria argyraea* | Fabaceae | Agricultural Research Council, 2016 |
|  | *Crotalaria dinteri* | Fabaceae | Agricultural Research Council, 2016 |
|  | *Crotalaria podocarpa* | Fabaceae | Agricultural Research Council, 2016; Eardley, 2012 |
|  | *Crotolaria virgultalis* | Fabaceae | Agricultural Research Council, 2016; Eardley, 2012 |
|  | *Crotalaria* sp. | Fabaceae | Agricultural Research Council, 2016; Gess and Gess, 2014 |
|  | *Gomphocarpus filiformis* | Asclepiadaceae | Gess and Gess, 2014 |
|  | *Monechma genistifolium* | Acanthaceae | Agricultural Research Council, 2016 |
|  | *Monechma mollissimum* | Acanthaceae | Gess and Gess, 2014 |
|  | *Monechma spartioides* | Acanthaceae | Agricultural Research Council, 2016 |
|  | *Monechma* sp. | Acanthaceae | Agricultural Research Council, 2016; Eardley, 2012; Gess and Gess, 2014 |
|  | *Polygala leptophylla* | Polygalaceae | Agricultural Research Council, 2016 |
|  | *Sesamum triphyllum* | Pedaliaceae | Agricultural Research Council, 2016; Gess and Gess, 2014 |
|  | *Sesamum* sp. | Pedaliaceae | Agricultural Research Council, 2016; Eardley, 2012; Gess and Gess, 2014 |
|  | *Tephrosia oxygona* | Fabaceae | Agricultural Research Council, 2016; Gess and Gess, 2014 |
|  | - | Acanthaceae | Gess and Gess, 2014 |
|  | - | Apocynaceae | Gess and Gess, 2014 |
|  | - | Asteraceae | Single male specimen, Gess and Gess, 2014 |
|  | - | Brassicaceae | Gess and Gess, 2014 |
|  | - | Fabaceae | Gess and Gess, 2014 |
|  | - | Pedaliaceae | Gess and Gess, 2014 |
|  | - | Polygalaceae | Gess and Gess, 2014 |
| *Megachile niveofasciata* | *Asclepias buchenaviana* | Asclepiadaceae | Agricultural Research Council, 2016 |
|  | *Aspalathus chortophila* | Fabaceae | Agricultural Research Council, 2016 |
|  | *Berkheya* sp. | Asteraceae | Eardley, 2012 |
|  | *Cleome paxii* | Cleomaceae | Agricultural Research Council, 2016 |
|  | *Geigeria* sp. | Asteraceae | Agricultural Research Council, 2016 |
|  | *Hermannia modesta* | Malvaceae | Agricultural Research Council, 2016 |
|  | *Lebeckia multiflora* | Fabaceae | Agricultural Research Council, 2016 |
|  | *Lessertia macrostachya* | Fabaceae | Agricultural Research Council, 2016 |
|  | *Limeum fenestratum* | Molluginaceae | Agricultural Research Council, 2016 |
|  | *Maerua gilgii* | Capparaceae | Agricultural Research Council, 2016 |
|  | *Maerua schinzii* | Capparaceae | Gess and Gess, 2014 |
|  | *Pentzia sphaerocephala* | Asteraceae | Agricultural Research Council, 2016 |
|  | *Polymita albiflora* | Aizoaceae | Agricultural Research Council, 2016; Eardley, 2012 |
|  | *Prenia pallens* | Aizoaceae | Agricultural Research Council, 2016 |
|  | *Psilocaulon salicornioides* | Aizoaceae | Agricultural Research Council, 2016 |
|  | *Sesamum triphyllum* | Pedaliaceae | Agricultural Research Council, 2016 |
|  | *Sisyndite spartea* | Zygophyllaceae | Eardley, 2012 |
|  | *Zygophyllum simplex* | Zygophyllaceae | Agricultural Research Council, 2016 |
|  | *Wiborgia* sp. | Fabaceae | Agricultural Research Council, 2016; Eardley, 2012 |
|  | *-* | Mesembryanthemaceae | Agricultural Research Council, 2016 |
| *Megachile venusta* | *Acacia caffra* | Fabaceae | Agricultural Research Council, 2016; Eardley, 2013 |
|  | *Acacia karroo* | Fabaceae | Agricultural Research Council, 2016 |
|  | *Allium* sp. | Amaryllidaceae | Agricultural Research Council, 2016; Eardley, 2013 |
|  | *Aptosimum procumbens* | Scrophulariaceae | Agricultural Research Council, 2016; Eardley, 2013 |
|  | *Aspalathus subtingens* | Fabaceae | Agricultural Research Council, 2016; Eardley, 2013 |
|  | *Bergia glomerata* | Elatinaceae | Agricultural Research Council, 2016 |
|  | *Bulbine frutescens* | Asphodelaceae | Agricultural Research Council, 2016 |
|  | *Eucalyptus* sp. | Myrtaceae | Agricultural Research Council, 2016; Eardley, 2013 |
|  | *Foeniculum vulgare* | Apiaceae | Agricultural Research Council, 2016 |
|  | *Grewia occidentalis* | Malvaceae | Agricultural Research Council, 2016; Eardley, 2013 |
|  | *Lycium* sp. | Solanaceae | Agricultural Research Council, 2016; Eardley, 2013 |
|  | *Medicago sativa* | Fabaceae | Agricultural Research Council, 2016; Eardley, 2013 |
|  | *Melolobium candicans* | Fabaceae | Agricultural Research Council, 2016 |
|  | *Polygala pinifolia* | Polygalaceae | Agricultural Research Council, 2016; Eardley, 2013 |
|  | *Portulaca* sp. | Portulacaceae | Agricultural Research Council, 2016; Eardley, 2013 |
|  | *Prosopis chilensis* | Fabaceae | Agricultural Research Council, 2016; Eardley, 2013 |
|  | *Rhus* sp. | Anacardiaceae | Agricultural Research Council, 2016; Eardley, 2013 |
|  | *Tribulus* sp. | Zygophyllaceae | Agricultural Research Council, 2016; Eardley, 2013 |
|  | *Trifolium* sp. | Fabaceae | Agricultural Research Council, 2016; Eardley, 2013 |
|  | *Vernonia* sp. | Asteraceae | Agricultural Research Council, 2016; Eardley, 2013 |
|  | *Vicia faba* | Fabaceae | Agricultural Research Council, 2016; Eardley, 2013 |
|  | *Zea mays* | Poaceae | Agricultural Research Council, 2016; Eardley, 2013 |
|  | - | Amaranthaceae | Goosefoot family Chenopodiaceae species observed, Agricultural Research Council, 2016; Eardley, 2013 |
|  | - | Asteraceae | Agricultural Research Council, 2016; Eardley, 2013 |
|  | - | Brassicaceae | Agricultural Research Council, 2016; Eardley, 2013 |
|  | - | Fabaceae | Agricultural Research Council, 2016 |
|  | - | Iridaceae | Agricultural Research Council, 2016; Eardley, 2013 |
|  | - | Liliaceae | Agricultural Research Council, 2016; Eardley, 2013 |
|  | - | Loganiaceae | Agricultural Research Council, 2016; Eardley, 2013 |
|  | - | Mesembryanthemaceae | Agricultural Research Council, 2016; Eardley, 2013 |
|  | - | Myrtaceae | Agricultural Research Council, 2016; Eardley, 2013 |
|  | - | Poaceae | Agricultural Research Council, 2016; Eardley, 2013 |
|  | - | Solanaceae | Agricultural Research Council, 2016; Eardley, 2013 |

Agricultural Research Council, 2016. Catalogue of Afrotropical Bees. doi:10.15468/u9ezbh

Eardley, C., 2013. A taxonomic revision of the southern African leaf-cutter bees, *Megachile* Latreille *sensu stricto* and *Heriadopsis* Cockerell (Hymenoptera: Apoidea: Megachilidae). Zootaxa 3601. doi:10.11646/zootaxa.3601.1.1

Eardley, C.D., 2012. A taxonomic revision of the southern African species of dauber bees in the genus *Megachile* Latreille (Apoidea:Megachilidae). Zootaxa 3460, 1–139.

Gess, S.K., Gess, F.W., 2014. Wasps and bees in Southern Africa. South African National Biodiversity Institute, Pretoria.
