# Supplementary material for: Floral hosts of leaf-cutter bees (Megachilidae) in a biodiversity hotspot revealed by pollen DNA metabarcoding of historic specimens
Source: PLoS One. 2021 Jan 21;16(1):e0244973. doi: 10.1371/journal.pone.0244973 (PMC7819603; doi:10.1371/journal.pone.0244973)
Supplement: S1 Fig — The lines for all samples collected from specimens from all six of the bee species flattened when 1,000 sequence reads were reached, indicating that all possible plant families represented in the pollen samples have been identified. a) and b) represents bee species occurring in the Savanna biome, c) and d) represents bee species occurring exclusively in the Succulent Karoo biome, and e) and f) represents bee species occurring all over South Africa (cosmopolitan group). (DOCX) [file pone.0244973.s008.docx]

**S1 Fig. Rarefaction curves for each bee species to determine whether sequence saturation has been reached with the amount of reads sequenced per pollen sample.** The lines for all samples collected from specimens from all six of the bee species flattened when 1,000 sequence reads were reached, indicating that all possible plant families represented in the pollen samples have been identified. a) and b) represents bee species occurring in the Savanna biome, c) and d) represents bee species occurring exclusively in the Succulent Karoo biome, and e) and f) represents bee species occurring all over South Africa (cosmopolitan group).
