# Supplementary material for: Floral hosts of leaf-cutter bees (Megachilidae) in a biodiversity hotspot revealed by pollen DNA metabarcoding of historic specimens
Source: PLoS One. 2021 Jan 21;16(1):e0244973. doi: 10.1371/journal.pone.0244973 (PMC7819603; doi:10.1371/journal.pone.0244973)
Supplement: Attachment — Submitted filename: Response to Reviewers_.docx [file pone.0244973.s009.docx]

PONE-D-20-18859
Floral hosts of leaf-cutter bees (Megachilidae) in a biodiversity hotspot revealed by pollen DNA metabarcoding of historic specimens
PLOS ONE

Dear Prof Smagghe (Academic Editor) and PLOS ONE editorial team,

Thank you for allowing us to resubmit a revised version of our manuscript. We have carefully gone through the issues raised by the reviewers and have addressed each below.

All the data generated in the study has been uploaded to FigShare and has been made public. The DOI are as follows: : *M. feline* (https://doi.org/10.6084/m9.figshare.12994067); *M. karooensis* (https://doi.org/10.6084/m9.figshare.12994115); *M. maxillosa* (https://doi.org/10.6084/m9.figshare.12994148); *M. murina* (https://doi.org/10.6084/m9.figshare.12994223); *M. niveofasciata* (https://doi.org/10.6084/m9.figshare.12994238). Data for *M. venusta* are available in the European Nucleotide Archive (ENA) under project accession PRJEB14178 (<http://www.ebi.ac.uk/ena/data/view/PRJEB14178>). Please can the “Data Availability Statement” be updated with this information.

We hope that this manuscript now meets the standards required for publication in PLOS ONE. If you require any additional information please do not hesitate to contact me using the contact details provided during submission.

kind regards

Dr Willows-Munro (corresponding author on behalf of all authors)

1. Please ensure that your manuscript meets PLOS ONE's style requirements, including those for file naming.

*We have changed the file names and manuscripts now meets style requirements.*

 2. We note that you have stated that you will provide repository information for your data at acceptance. Should your manuscript be accepted for publication, we will hold it until you provide the relevant accession numbers or DOIs necessary to access your data. If you wish to make changes to your Data Availability statement, please describe these changes in your cover letter and we will update your Data Availability statement to reflect the information you provide.

*We have deposited the data in FigShare and have included the DOIs in the cover letter.*

3. Please include a copy of Table 3 which you refer to in your text on page 12.

*We removed Table 3 from the manuscript during preparation as the information is replicated in Figure 2. We left this citation in by mistake, this has been corrected.*

Reviewers' comments:
Reviewer #1: Gous et al. report on the use of pollen barcoding on samples from a historic museum collection to test differences in floral host range of different Megachile spp. More specifically they test if Megachile spp. with a widespread geographical range (in South Africa) are more generalist compared to species with a more confined geographical range. The manuscript is well written and technically sound, yet I do mis the novelty and clear message of the manuscript. They state several times that the study shows that historic insect specimens can be used, yet they showed this already in their previous article with even older samples. The study indeed shows that host range can be deduces using historical samples, yet this is not really ‘novel’. This being said the authors pose an interesting hypothesis, with regard to pollinator specificity, which I think is worth further investigation using a wider range of species and a higher number of samples (keeping in mind the limitation of historic samples) to make a stronger claim.

*We have thoroughly edited the manuscript so that the message is clearer. In particular we place more emphasis on the use of metabarcoding to investigate pollinator specificity. Although we agree that sampling a wide range of species would provide interesting comparison, this is beyond the scope of the present study. The data was generated as part of Gous PhD study which concluded in 2018. Although our sample size was limited by using historical samples, the number of samples used is comparable to that used in many recent studies using metabarcoding of pollen.*

Reviewer #2: This article is very relevant in the context of biodiversity conservation, and as stated in the conclusion, can give more understanding of possible impact of climate change on endemism. This 'search to understanding' was lacking in the discussion of the paper; the presence of plant families in the pollen loads was only explained through comparison with observation data or other literature, but not in context of ecology and possible effects on shifts in plant-bee interactions due to climate change. But to answer those type of questions, a lot more data is needed, on many different bee species. Also, historical data will not give much explanation to current effects of climate change.

*Agreed, answering these important multi-species questions such as shifts in plant-bee interactions over time will require many years of work and is beyond the scope of this study. Here we focus on the application of metabarcoding to study pollination specificity in a group of indigenous bees from a biodiversity hotspot. Our data is publicly available and we hope will be useful in future to conduct such large-scale meta-analyses.*

The choice to work with only Megachilidae and 2 species per group (biome or widespread), is understandable but does not give opportunity to answer any questions more than 'Is metabarcoding from historic specimens possible and how does it compare to observational data'. The sentence on line 435-437 is thus not suitable and also ambiguous.

*We have rephrased the sentence to better fit the aim of our study. Our data does provide evidence that species with narrower distributions and pollination specification are more likely to be impacted by climate change that species that are more widespread.*

The hypothesis on line 346- 351 (and 96) is not very clear. The link between high levels of floral endemism and species richness should be more grounded.
On line 346, a first hypothesis is explained, but the second hypothesis is not stated anywhere.

*This sentence has been rephrased for clarification.*

Some other questions I had were:
- 25: what are the three globally recognised biodiversity hotspots and are they used/relevant to this study?

*The three biodiversity hotspots are: the Cape Floristic Region; the Succulent Karoo; and the Maputaland-Pondoland-Albany hotspot. Our study did not focus specifically on bees in these three regions. The mention of the three hotspots was to justify the statement that South Africa is characterized by exceptionally high biodiversity.*

- 75: what is meant by susceptibility?

*We have changes this to “sensitive”.*

- 95: why the choice of only looking at Megachilidae, and if relevation explanation; add in text

*We chose this group because the family contains both species endemic to a single biome (endemic to either western South Africa or eastern South Africa) and species that are widespread. There is also a fair amount of observational data available on members of this family. Most importantly, we have a large historical collection for the family. Few other families are as well represented in the national collection.*

- 110: how and why were these 6 species chosen?

*See above. Mostly these species were chosen based on the availability of historical material.*

- 168: why the use of HiSeq when MiSeq was used in a previous study? Will this not affect species outcome because of different sequencing depths?

*The Miseq data was generated in a previous study (Gous et al. 2019). In this study we had the Hiseq available at the Agricultural Research Council sequencing platform and decided to use it. In all sequencing runs we determined that the sequencing depth was sufficient to capture the pollen diversity and so we feel that combining data from two different sequencers has not biased the results.*

- 369 until 387:on what is this knowledge about tribes based? the 6 species studied were all from the same tribe?

*I don’t really understand this comment, we have provided references to support our statements in the text.*
