# Supplementary material for: Floral hosts of leaf-cutter bees (Megachilidae) in a biodiversity hotspot revealed by pollen DNA metabarcoding of historic specimens
Source: PLoS One. 2021 Jan 21;16(1):e0244973. doi: 10.1371/journal.pone.0244973 (PMC7819603; doi:10.1371/journal.pone.0244973)
Supplement: Attachment — Submitted filename: Response to reviewers.docx [file pone.0244973.s010.docx]

PONE-D-20-18859
Floral hosts of leaf-cutter bees (Megachilidae) in a biodiversity hotspot revealed by pollen DNA metabarcoding of historic specimens
PLOS ONE

Dear Prof Smagghe (Academic Editor) and PLOS ONE editorial team,

Thank you for allowing us to respond to the comment raised by Reviewer 1. In this study we use data from multiple individuals belonging to six species of Megachile bees, which differ in habitat specificity. We feel that the data collected is appropriate for the hypothesis tested and our conclusions are supported by the data. We do not feel that adding additional data to the study will significantly change the conclusions. We received a very favourable review from Reviewer 2, who noted that this is a ”valuable contribution to important biodiversity data”. The data presented in this study was generated during a PhD. The student has completed her studies and there is no opportunity to generate more data. We are thus unable to add more samples to our study, but feel that our manuscript is strong enough for publication in PLOS ONE given the very favourable review we received from Reviewer 2. We have made no changes to the manuscript, but resubmit “as is” for consideration.

We hope that this manuscript now meets the standards required for publication in PLOS ONE. If you require any additional information please do not hesitate to contact me using the contact details provided during submission.

kind regards

Dr Willows-Munro (corresponding author on behalf of all authors)
